# Sea Urchin-like Magnetic Microbeads-Based Electrochemical Biosensor for Highly Sensitive Detection of Metabolites

**DOI:** 10.3390/bios15040225

**Published:** 2025-04-02

**Authors:** Bin Chen, Xiaosu Yuan, Enze Tian, Yunjie Tan, Le Li, Ru Huang

**Affiliations:** 1 State Key Laboratory of Digital Medical Engineering, Key Laboratory of Biomedical Engineering of Hainan Province, School of Biomedical Engineering, Hainan University, Sanya 572024, China; 2 NHC (National Health Commission of the People’s Republic of China) Key Laboratory of Tropical Disease Control, Hainan Medical University, Haikou 571199, China

**Keywords:** sea urchin-like microbeads, magnetic microbeads, metabolite detection, electrochemical biosensor

## Abstract

Analyzing metabolite levels in bodily fluids is essential for disease diagnosis and surveillance. Electrochemical biosensors are ideal for monitoring metabolite levels due to their high sensitivity, rapid response, and low cost. The magnetic microbeads-based electrode functionalization method further promotes the automation development of electrochemical biosensors by eliminating the tedious electrode polishing process. In this study, we presented sea urchin-like magnetic microbeads (SMMBs) and constructed an SMMB-based electrochemical biosensor. The specific morphology of SMMBs provides a larger specific surface area and abundant enzyme binding sites, thereby expanding the active reaction interface on the electrode and improving the sensitivity of the biosensor. Experiment results demonstrated that the SMMB-based electrochemical biosensor achieves μM level detection sensitivity for glucose. Furthermore, by replacing the anchored oxidase on SMMBs, the biosensor can be extended to detect other metabolites, such as cholesterol. In summary, the SMMBs provide a new path to handily construct electrochemical biosensors and hold a great potential for metabolite detection and further development.

## 1. Introduction

A large number of metabolites, such as glucose, cholesterol (Chot), ʟ-lactate, and xanthine, etc., have been found to play critical roles in metabolic disorders and associated diseases [1,2,3,4]. Monitoring metabolite levels in bodily fluids is essential for controlling disease progression and testing the pharmacological responses to therapeutics [5,6,7]. For instance, abnormal blood glucose levels are strongly related to the development of diabetes [8]. Maintaining glycemia within a relatively normal range is the most effective method for diabetes self-management [9,10]. As the incidence rate of diabetes increases year by year [11], glucose sensors now account for 85% of the biosensor market, improving the quality of life for diabetic patients greatly [12,13,14]. Additionally, other metabolic diseases, including obesity, gout, and lactic acidosis, underscore the urgent need for convenient and effective biosensors to detect corresponding metabolic biomarkers [15,16,17,18,19].

Electrochemical biosensors are among the most widely used sensors valued for their advantages of high sensitivity, rapid response, low cost, and, most notably, their ease of miniaturization and automation [20,21,22,23,24,25]. To enhance the performance and broaden the utility of electrochemical biosensors, various smart nanomaterials have been introduced into these systems [26,27]. For instance, magnetic nanoparticles functionalized with target recognition elements can be guided to the surface of the working electrode using a magnetic field [28,29]. This approach avoided the labor-intensive electrode polishing step, significantly simplifying the operation and enabling greater automation of electrochemical biosensors [30,31]. In addition, nanocomposites are frequently used as carriers for target recognition elements or signal tags, for their large specific surface area offers abundant binding sites [32,33].

In this study, we synthesized sea urchin-like Fe_3_O_4_@ZnO magnetic microbeads (SMMBs) and constructed an SMMB-based electrochemical biosensor for the detection of metabolites, including glucose and Chot. Zinc oxide (ZnO), a common and environment-friendly semiconductor material, was chosen to construct the sea urchin-like surface of SMMBs. It has been reported that rod-like ZnO nanomaterials have good electrocatalytic performance and anti-interference ability, which supports their application in the analysis of complex samples [34,35,36,37,38]. Also, the positively charged surface of SMMBs enables efficient and convenient anchoring of oxidases by electrostatic interaction. Moreover, the sea urchin-like morphology provides SMMBs with a large specific surface area and abundant oxidase binding sites, which could greatly improve the sensitivity of the biosensor. Furthermore, the magnetism of SMMBs endows the electrochemical biosensor with great flexibility and simplicity, making it suitable for the analysis of metabolites.

## 2. Materials and Methods

### 2.1. Materials

C_4_H_6_O_4_Zn·2H_2_O (ZnAc), KCl, K_3_FeC_6_N_6_, K_4_FeC_6_N_6_·3H_2_O, C_2_H_6_O_2_, and polyethyleneimine (PEI, MW 1800, 99%) were purchased from Aladdin (Shanghai, China). NaCl, NaOH, polyethylene glycol (PEG, average Mn 4000), Na_2_HPO_4_ (anhydrous), FeCl_3_·6H_2_O, Chot, cholesterol oxidase (ChOx), CH_3_CH(OH)CH_3_, uric acid (UA), and ւ-lysine (ւ-Lys) were purchased from Macklin (Shanghai, China). Zn(NO_3_)_2_·6H_2_O, CH_3_CH_2_OH, and hexamethylenetetetramine (HMTA) were from Xilong Science. Triton TM X-100 was purchased from Sigma-Aldrich (Shanghai, China). C_6_H_5_O_7_Na_3_·2H_2_O, glucose oxidase (GOx), horseradish peroxidase (HRP), phosphate-buffered saline (PBS, 0.01 M), and glucose were purchased from Biochem (Shanghai, China). All electrodes used in this study were purchased from Shanghai Yingke Union. C_2_H_3_NaO_2_ (NaAc) and 2,2′-azino-bis(3-ethylbenzothiazoline-6-sulfonic) (ABTS) were purchased from Anegi (Shanghai, China). Bovine serum albumin (BSA) was purchased from Sangon (Shanghai, China).

The apparatus for microbeads characterization includes a scanning electron microscope (SEM, Crossbeam 350, Zeiss, Oberkochen, Germany), transmission electron microscope (TEM, FEI Talos F200X, Thermo Fisher, Waltham, MA, USA), Zetasizer Pro (Malvern Panalytical Ltd., Malvern, UK), and microplate reader (SpectraMax iD5, MolecularDevices, San Jose, CA, USA).

### 2.2. Preparation of Fe_3_O_4_ Magnetic Microbeads (MMBs), Urchin-like Fe_3_O_4_@ZnO Magnetic Microbeads (SMMBs), and SMMB/Oxidase

First, 2.70 g of FeCl_3_·6H_2_O, 0.40 g of C_6_H_5_O_7_Na_3_·2H_2_O, and 0.5 g of PEG were dissolved in 70 mL of ethylene glycol. Once completely dissolved, 3.854 g of NaAc was added, and the mixture was stirred for 30 min, followed by heating to 200 °C for 24 h. The products were collected by using a magnet and washed several times with anhydrous ethanol.

Second, MMB@ZnO was prepared by dissolving 55.0 mg ZnAc and 10 mg NaOH in 50 mL of anhydrous ethanol. Subsequently, 6 mg of MMBs were dispersed into the solution, followed by sonication for 5 min, mechanically stirring for 12 h, and annealing at 150 °C for 2 h. The products were washed with deionized water.

Then, 297.4 mg of Zn(NO_3_)_2_·6H_2_O, 140.2 mg of HMTA, and 72.0 mg of PEI were dissolved in 40 mL of deionized water. MMB@ZnO was added to the solution, sonicated for 5 min, and heated at 90 °C for 2 h. The products were washed with deionized water to remove unreacted components.

GOx/ChOx-anchored SMMBs were obtained by incubating GOx or ChOx with SMMBs for 30 min. The oxidase adsorption efficiency of the microbeads was analyzed using a BCA Protein Assay Kit (Sangon, Shanghai). The collected SMMBs/oxidase were washed three times with sterile water and stored at 4 °C for further use. The activity of the oxidase anchoring on the SMMBs was evaluated by an HRP-catalyzed chromogenic reaction of ABTS. Detailed experimental procedures for characterization of the microbeads are shown in Supplementary Materials (Section S1).

### 2.3. Electrochemical Detection of Metabolites

The electrochemical biosensor was composed of a magnetic glassy carbon electrode (MGCE) as the working electrode (WE), a platinum electrode as the counter electrode, and an Ag/AgCl electrode as the reference electrode. The voltage–current characteristics were tested in 3 mL 5 mM [Fe(CN)_6_]^3−/4−^ solution, which was prepared by adding 1 mL 15 mM K_3_FeC_6_N_6_ and 1 mL 15 mM K_4_FeC_6_N_6_·3H_2_O into 0.01 M PBS.

The performance of the SMMB/GOx-based electrochemical biosensor for glucose detection was determined by chronoamperometry. First, 20 μL 2 mg/mL SMMB/GOx was dropped onto the surface of the WE, and then the WE was slowly inserted into 0.01 M PBS. The current signal was recorded by using an electrochemical workstation (CHI1030c, CH Instruments, Austin, TX, USA). The applied potential was 1 V (vs. Ag/AgCl electrode).

The Chot solution was prepared by mixing isopropanol, Triton TM X-100, and PBS in a volume ratio of 1:1:3. The detection procedure was the same as that used in the glucose assay. The performance of the SMMB/ChOx-based electrochemical biosensor for Chot detection was determined by cyclic voltammetry (CV) with a scan rate of 50 mV/s over a potential range of 0 V to +1.3 V.

## 3. Results

### 3.1. Preparation of SMMBs

**Figure 1:**
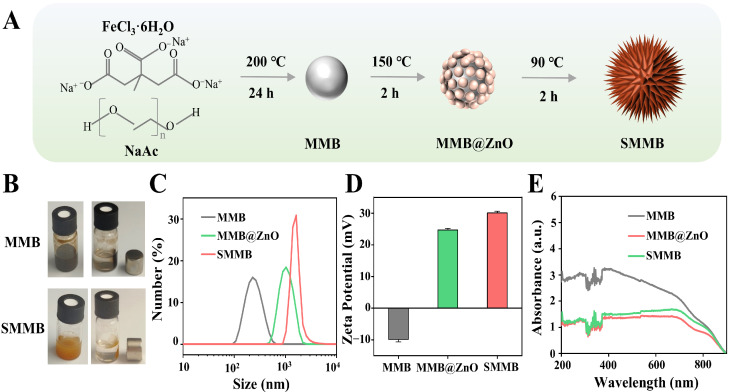
Preparation and characterization of SMMBs. (**A**) The preparation method and process of SMMBs. (**B**) The photographs of MMB and SMMB solutions. When a magnet was placed near the bottle, both MMBs and SMMBs quickly formed a precipitate on the side near the magnet; meanwhile, the solution became clear. (**C**) The particle size analysis results. (**D**) ζ–potential analysis results. The bars display the mean values ± standard error from three replicates. (**E**) The absorbance analysis results of MMBs, MMB@ZnO, and SMMBs, respectively.

The synthesis process of SMMBs is illustrated in Figure 1A. The obtained products, the MMB and SMMB solutions, are shown in Figure 1B. As seen, the MMB solution appears black, while the SMMB solution shows a brownish-yellow color. Both MMBs and SMMBs can be rapidly adsorbed by a magnet, which confirms their strong magnet responsiveness. The analysis results of particle size and ζ-potential (Figure 1C,D) indicate that the hydrodynamic diameter of the MMB is 242.2 ± 21.59 nm, and its ζ-potential is −9.85 ± 0.76 mV. In comparison, the hydrodynamic diameter and ζ-potential of the MMB@ZnO are 1095 ± 98.1 nm and 24.7 ± 0.54 mV, respectively, while those of SMMBs are 1768 ± 153 nm and 30.1 ± 0.5 mV, which confirmed that ZnO has been deposited onto the MMBs. In addition, three microbeads, MMBs, MMB@ZnO, and SMMBs, were analyzed by UV-Vis absorption spectroscopy (Figure 1E). All three microbeads exhibit obvious absorbance within 200~800 nm. The deposition of ZnO caused a significant decrease in the absorbance value. The absorbance spectra of the MMB@ZnO and SMMBs show no significant difference because the composition of the MMB@ZnO and SMMBs have no essential difference, though their morphologies are quite different.

### 3.2. Characterization of SMMBs

**Figure 2:**
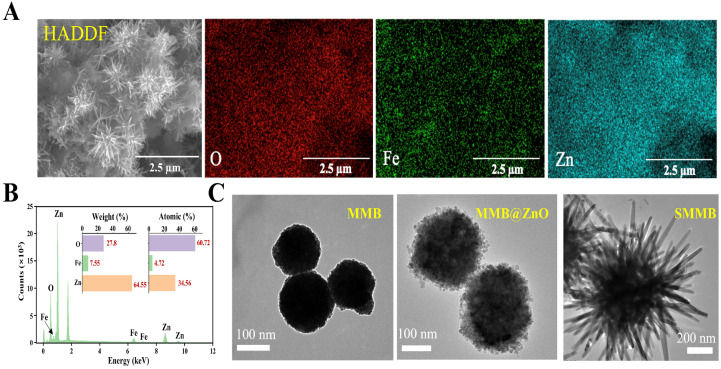
Characterization of the SMMBs. (**A**) HADDF-STEM brightfield image of SMMBs, along with elemental mapping of oxygen (O), iron (Fe), and zinc (Zn). The scale bars are 2.5 μm. (**B**) EDS spectrum of SMMBs. The inset shows the mass and atomic percentage of O, Fe, and Zn. (**C**) TEM images of MMBs, MMB@ZnO, and SMMBs. The scale bars are 100 nm, 100 nm, and 200 nm, respectively.

To verify the morphology of the microbeads, high-angle annular dark field scanning transmission electron microscope (HADDF-STEM) and energy dispersive X-ray spectroscopy (EDS) elemental mapping are given in Figure 2A. It can be seen that SMMBs possess a uniform sea urchin-like morphology composed of O, Fe, and Zn. The corresponding EDS analysis result is shown in Figure 2B. Furthermore, the morphologies of MMBs, MMB@ZnO, and SMMBs were characterized using TEM (Figure 2C). As seen, MMBs exhibit a smooth spherical shape with an average diameter of ~160 nm, while MMB@ZnO displays a rougher surface and increased diameter (~250 nm) due to the ZnO coating, and SMMBs present an ideal sea urchin-like structure with an approximate overall diameter of ~1.2 μm. The thorns on the surface, measured from their base to tip, are about 430 nm in length. Moreover, X-ray diffraction (XRD) and high-resolution transmission electron microscopy (HR-TEM) were used to analyze the crystal structure and the atomic-scale structure of the SMMBs, respectively (Figures S1 and S2). All of these results can indicate that SMMBs have been successfully constructed.

### 3.3. Preparation of SMMB-Based Electrochemical Biosensor for Glucose Detection

**Figure 3:**
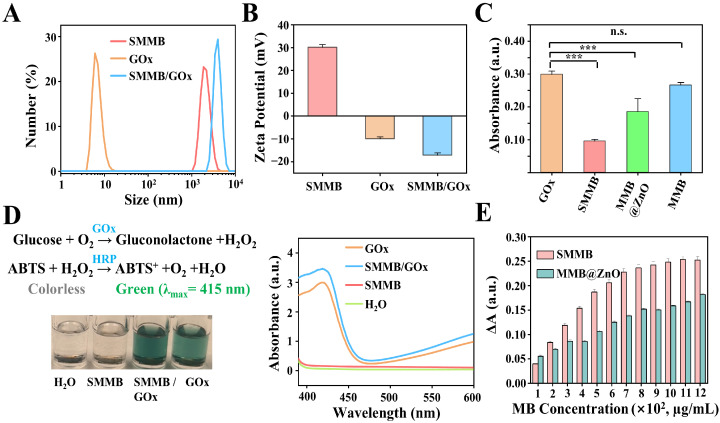
Preparation and characterization of SMMB-based electrochemical biosensor. The hydrodynamic diameter (**A**) and ζ-potential (**B**) analysis results of SMMBs, GOx, and SMMB/GOx, respectively. (**C**) Comparison of the GOx adsorption ability of different microbeads (*t*-test; n.s., not significan; *** *p* < 0.001. n = 3). (**D**) Analysis of the SMMB-anchored GOx activity. Colorless ABTS was used as a substrate of the chromogenic reaction that can be oxidized by H_2_O_2_ to green ABTS^+^ under the catalysis of HRP. The corresponding absorbance detection results are shown in the nether. (**E**) The ΔA was obtained by subtracting the absorbance of each sample from the absorbance of the GOx (500 μg/mL). All plots show mean ± SD for n = 3 replicates.

To construct an SMMB-based electrochemical biosensor, the first step is to anchor the oxidase onto the surface of the SMMB. Glucose, a widely used biomarker for diabetes [39,40], was selected as a model target. Therefore, GOx was anchored on the SMMB surface to mediate specific glucose recognition and signal transduction. First, the analysis results of particle sizes and ζ-potential in Figure 3A,B indicated that after being absorbed by GOx, the hydrodynamic diameter of the SMMBs increased to 2800 ± 600 μm, and the ζ-potential changed to −25 ± 1.5 mV, demonstrating effective adsorption of GOx onto the SMMBs.

The adsorption efficiency of the microbeads for GOx was quantified by testing the activity of the remaining GOx in the supernatants (Supplementary Material S1). The results (Figure 3C and Figure S3) displayed that both SMMBs and MMB@ZnO effectively adsorbed negatively charged GOx through their ZnO-based positively charged surface, resulting in a significant decrease in the absorbance of their supernatants. In comparison, the bare MMBs, with their negatively charged surface, exhibited negligible adsorption of GOx. Additionally, the larger specific surface area of SMMBs provides more binding sites for GOx, thereby enabling it to adsorb more GOx than MMB@ZnO.

Next, the catalytic activity of the GOx on the surface of the SMMBs was analyzed. The reaction principle and the experimental result are shown in Figure 3D. It showed that both GOx and SMMB/GOx effectively catalyzed the color reaction of ABTS and induced the emergence of a distinct absorption peak at ~415 nm, confirming that the SMMB-anchored GOx maintained catalytic activity as high as the dissociative GOx.

Then, the microbeads (SMMBs, MMB@ZnO, and MMBs) were added onto MGCE, respectively, and the voltage–current characteristics were tested in 5 mM of [Fe(CN)_6_]^3−/4−^ solution with a scan rate of 0.1 V/s. As shown in Figure S5, the bare MGCE displayed the highest current peak, while SMMBs and MMB@ZnO showed relatively weak current peaks due to the semiconductor nature of ZnO. MMBs showed the lowest current peak because Fe_3_O_4_ possesses weaker conductivity than ZnO.

The amount of GOx on the SMMB-functionalized MGCE is a key parameter influencing the detection performance of the biosensor since an insufficient amount of GOx will result in a weak detectable signal. To optimize the loading amount of GOx, a series of SMMBs and MMB@ZnO samples with different concentrations were mixed with 500 μg/mL GOx. After magnet separation, the amount of the remaining GOx in supernatants was quantified by BCA assay (Figure S6). The further comparison results in Figure 3E obviously displayed that SMMBs hold significantly higher GOx absorption efficiency than MMB@ZnO. Also, it revealed that about 800 μg/mL SMMBs fully adsorbed 500 μg/mL GOx, which was used as the optimal ratio for the following experiments.

### 3.4. Analyzing the Performance of the SMMB-Based Electrochemical Biosensor for Glucose Detection

**Figure 4:**
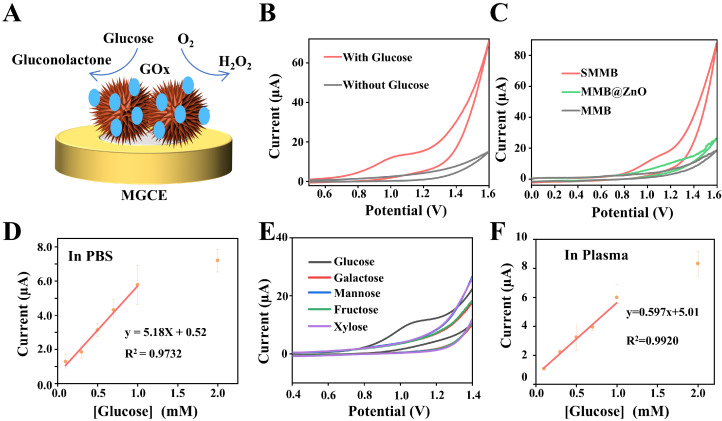
The performance of the SMMB-based electrochemical biosensor for glucose detection. (**A**) The diagram of the SMMB/GOx mediated glucose oxidation reaction on the MGCE. (**B**) The feasibility of the SMMB-based electrochemical biosensor for glucose detection. The glucose concentration was 2.5 mM (**C**) Comparison of the performance of the SMMB-, MMB@ZnO-, and MMB-based biosensor for glucose detection, respectively. The concentrations of the GOx and the microbeads were 500 and 800 μg/mL, respectively. The glucose concentration was 1.25 mM. (**D**) Analysis of standard curves for the ability of electrochemical biosensor-based SMMBs to detect different concentrations of glucose. (**E**) Selectivity analysis of the SMMB-based electrochemical biosensor. All of the monosaccharides are 2.5 mM. (**F**) Analyzing the detection ability of the SMMB-based electrochemical biosensor for different concentrations of glucose in 100× diluted plasma. All plots show mean ± SD for n = 3 replicates.

First, GOx was anchored onto SMMBs by electrostatic adsorption, forming SMMB/GOx. Then, the SMMB/GOx was added to the surface of the magnetic glassy carbon electrode (MGCE). On the electrode, glucose underwent oxidation to gluconolactone; meanwhile, O_2_ was reduced to H_2_O_2_ (Figure 4A). The current signal generated by this redox reaction was recorded. As a result, the intensity of the current signal was proportional to the glucose concentration.

Next, the feasibility of the SMMB-based electrochemical biosensor for glucose detection was tested. As shown in Figure 4B, the sample with 2.5 mM glucose displayed a higher current signal than the control sample without glucose, indicating the certain potential of the SMMB-based electrochemical biosensor for glucose detection. Also, GOx-functionalized SMMBs, MMB@ZnO, and MMBs were added onto the MGCE respectively, and their glucose detection ability were compared. The detection results in Figure 4C show that the SMMB-anchored electrode generated the strongest current signal, which may be attributed to the higher GOx loading capacity of SMMBs, enabled by its larger specific surface area and the efficient electrostatic interactions facilitated by its positively charged ZnO surface. In addition, the effect of electrolyte pH on CV response was tested. As shown in Figure S7, pH in the range of 6–8 had no significant effect on the CV profile of the test system. Considering the optimal reaction condition of GOx, PBS (pH 7.5) was used to perform the following experiments.

Then, the performance of the SMMB-based electrochemical biosensor for glucose detection was evaluated by adding a series of glucose with different concentrations (0, 0.1, 0.3, 0.5, 0.7, 1, 2 mM) into the system. The detection results in Figure S8 displayed that the current signal increased proportionally with the growth of glucose concentration. The analysis results in Figure 4D showed that as low as 0.1 mM glucose can be detected, and the linear range is from 0.1 to 1 mM. In addition, to test the selectivity of the SMMB-based electrochemical biosensor, several other monosaccharides, including galactose, mannose, fructose, and xylose, have been added into the electrolyte, respectively, and analyzed by the biosensor. As shown in Figure 4E, only glucose induced an apparent oxidation current peak, while other monosaccharides did not, which confirmed that the biosensor possesses good selectivity. Also, other non-target biomarkers, such as KCl, NaCl, ւ-Lys, and UA, were tested by the SMMB-based biosensor. The results (Figure S9) showed that the sample containing glucose induced the maximum current change, confirming the specificity for glucose detection of the SMMB-based electrochemical biosensor. Furthermore, the detection ability of the SMMB-based electrochemical biosensor for complex samples was evaluated by detecting glucose in diluted plasma. The results in Figure S10 showed that the biosensor reached a detection limit of 0.3 mM glucose, which is higher than those obtained in PBS buffer at equivalent glucose concentrations. It is likely attributed to the non-specific adsorption of plasma proteins, which inhibited electron transfer at the electrode surface. To tackle this problem, SMMB/GOx was treated with 5 mg/mL BSA to block the unmodified sites on the SMMBs surface and then was used to analyze glucose at different concentrations in diluted plasma. The detection results are shown in Figure 4F. The detection limit can reach 0.1 mM, which is consistent with the detection of glucose in buffer results. The results confirmed that protein in plasma may affect the performance of the biosensor, and suitable surface modification can effectively eliminate the interference. The performance of the SMMB-based biosensor was also compared with several existing nanomaterial-functionalized electrochemical sensors for glucose detection (Supplementary Material, Table S1).

### 3.5. Analyzing the Performance of the SMMB-Based Electrochemical Biosensor for Cholesterol Detection

**Figure 5:**
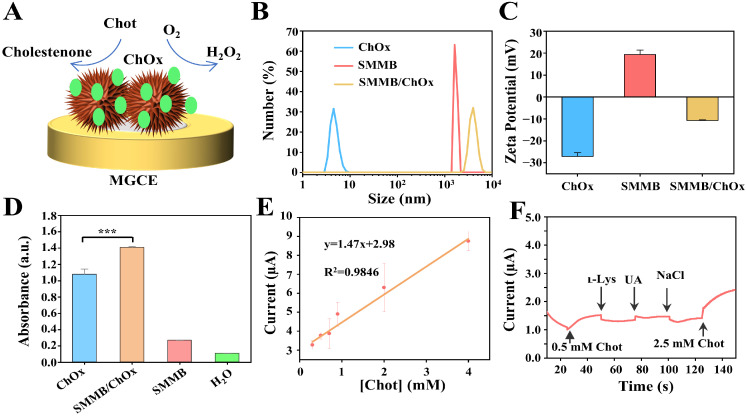
The performance of the SMMB-based electrochemical biosensor for Chot detection. (**A**) The diagram of the SMMB/ChOx mediated Chot oxidation reaction on the MGCE. The hydrodynamic diameter (**B**) and ζ-potential analysis results (**C**) of SMMBs, ChOx, and SMMB/ChOx, respectively. (**D**) Verification of the activity of ChOx on SMMBs by microplate reader (*t*-test; *** *p* < 0.001. n = 3). (**E**) Standard curve for the addition of different concentrations of Chot for detection sensitivity analysis. (**F**) Selectivity study of the SMMB/ChOx-based electrochemical biosensor in the presence of 0.5 mM Chot and 0.5 mM other interfering chemicals. The applied potential was 1 V. All plots show mean ± SD for n = 3 replicates.

The SMMB-based electrochemical biosensor is versatile and can detect other biomarkers by replacing the GOx with other oxidases. For instance, ChOx can be absorbed onto the surface of the SMMBs and catalyze Chot to form its oxidation products, cholestenone, on the MGCE, thereby enabling the detection of Chot concentration by monitoring the change of the current signal (Figure 5A). To verify the successful construction of the ChOx-functionalized SMMBs, hydrodynamic diameter and ζ-potential were measured by a Zetasizer. The results in Figure 5B,C show that the anchoring of ChOx led to an increase in hydrodynamic diameter of 4074 ± 613 nm, and ζ-potential changed from positive to negative (−10.7 ± 0.27 mV), indicating successful adsorption of ChOx onto SMMBs. Also, the activity of the ChOx anchoring on SMMBs was verified by measuring the change of the absorbance, which was caused by the HRP-catalyzed chromogenic reaction. The results in Figure 5D and Figure S11 show that both ChOx and SMMB/ChOx induced a remarkable absorption peak at 415 nm, which indicates SMMBs did not make a significant impact on ChOx activity. Next, the optimal SMMB to ChOx ratio for biosensor construction was determined by mixing different concentrations of SMMBs with 500 μg/mL ChOx and quantifying the dissociative ChOx with a BCA assay kit. As shown in Figure S12, nearly all ChOx was adsorbed by 125 mg/mL SMMBs. Therefore, the following experiments were performed using this optimized ratio.

Then, the performance of the SMMB/ChOx-based electrochemical biosensor was evaluated. The sensitivity for Chot detection was tested by adding Chot at different concentrations (0, 0.3, 0.5, 0.7, 0.9, 2, 4 mM) into the system, and the current signal was collected (Figure S13). The results showed that the SMMB/ChOx-based electrochemical biosensor achieved a detection limit of 0.3 mM with a linear range from 0.3 to 4 mM for Chot detection in Figure 5E. Moreover, the selectivity was also tested by using amperometric i-t to investigate the effect of several typical physiological chemicals (including ւ–Lys, UA, and NaCl; each of the chemicals were 0.5 mM) at an applied potential of 1 V. According to the detection result in Figure 5F, all of the amperometric current responses caused by non-target compounds were negligible, while the addition of 0.5 mM and 2.5 mM Chot caused significant current increase, which demonstrated the biosensor holds a good selectivity.

## 4. Conclusions

In summary, we prepared sea urchin-like Fe_3_O_4_@ZnO magnetic microbeads named SMMBs and constructed an SMMB-based electrochemical biosensor for the sensitive detection of metabolic biomarkers. Benefiting from the ZnO-based positively charged surface and the large specific surface area endowed by the sea urchin-like morphology, the SMMBs exhibited highly efficient oxidase adsorption ability and improved detection sensitivity. Moreover, the magnetic adsorption-based electrode functionalization mode avoided direct covalent modification or enzyme adsorption on the electrode, thereby avoiding a tedious electrode polishing process. According to the experiment results, the SMMB/GOx-based electrochemical biosensor demonstrated sub-mM level sensitivity for glucose detection. By replacing GOx with ChOx, the biosensor can be extended to Chot detection. However, the results also showed that the semiconductive ZnO shell of the SMMBs could prevent the electron transfer on the electrode surface [41,42,43,44,45], thereby limiting the further improvement of analytical sensitivity. Developing SMMBs with conductive shells would overcome this limitation and provide more sensitive electrochemical detection for metabolic biomarkers.

## Data Availability

The data supporting our article with the title “Sea urchin-like magnetic microbeads-based electrochemical biosensor for highly sensitive detection of metabolites” have been included as part of the ESI. Further information is available on request.

## Supplementary Materials

The following supporting information can be downloaded at https://www.mdpi.com/article/10.3390/bios15040225/s1, S1: Experimental methods for SMMB characterization. S2: Characterization of SMMBs with X-ray diffraction (XRD). S3: Characterization of SMMBs with high-resolution transmission electron microscopy (HR-TEM). S4: Analysis of the GOx adsorption ability of different microbeads by microplate reader. S5: Analysis of the anchoring of GOx on SMMBs by Fourier transform near-infrared spectroscopy (FTIR). S6: Cyclic voltammetry (CV) profiles for various microbeads modified electrodes. S7: Comparison of the GOx loading efficiency of MMB@ZnO and SMMBs. S8: Optimization of pH. S9: Performance of SMMB-based electrochemical biosensor for glucose detection. S10: Specificity analysis of SMMB-based electrochemical biosensors. S11: Analysis of the sensitivity of the SMMB-based biosensor for detecting glucose in plasma. S12: Analysis of the anchoring activity of ChOx on SMMBs. S13: Analysis of the ChOx loading efficiency of SMMBs; S14: The performance of the SMMB-based electrochemical biosensor for cholesterol (Chot) detection. Table S1: Existing electrochemical sensors for glucose detection. References [46,47,48,49,50] are cited in the supplementary materials.
