# Supplementary material for: Sea Urchin-like Magnetic Microbeads-Based Electrochemical Biosensor for Highly Sensitive Detection of Metabolites
Source: Biosensors (Basel). 2025 Apr 2;15(4):225. doi: 10.3390/bios15040225 (PMC12025299; doi:10.3390/bios15040225)
Supplement: Supplementary file 1 [file biosensors-15-00225-s001.zip › biosensors-3507091-supplementary.pdf]

biosensors

MDPI

Supplementary Material

Sea urchin-like magnetic microbeads based electrochemical biosensor for highly sensitive detection of metabolites

Bin Chen[1,‡], Xiaosu Yuan[1,‡], Enze Tian[1], Yunjie Tan[1], Le Li[2,*] Ru Huang[1,*]

[1] State key laboratory of Digital Medical Engineering, Key Laboratory of Biomedical Engineering of Hainan Province, School of Biomedical Engineering, Hainan University, Sanya, 572024, China.

[2] NHC(National Health Commission of the People's Republic of China) Key Laboratory of Tropical Disease Control, Hainan Medical University, Haikou, Hainan, P.R.China, 571199

‡ The authors contribute equally in this study.

* Correspondence: huangru@hainanu.edu.cn(R.H); leli@hainmc.edu.cn (L.L.).

S1 Experimental methods for SMMB characterization.

TEM imaging. The SMMB solution was diluted to 100 μg/mL and added onto a copper grid, overlaying it with a graphene film to improve image resolution. Before imaging, the sample was fully dried.

EDS testing. 10 μL of a 100 μg/mL SMMB solution was dropped onto a silicon wafer and dried with an infrared lamp. After that, the silicon wafer was attached to the sample stage with conductive adhesive for elemental analysis.

BCA assay. To compare the adsorption efficiency of SMMB, MMB@ZnO, and MMB for GOx, 800 μg/mL of each microbead type was incubated with 500 μg/mL GOx respectively. Then, the remaining GOx in the supernatants were quantified by a BCA protein quantification kit according to the protocol. In principle, the oxidase can complex with Cu^{2+} and reduce it to Cu^{+}, which reacts with bicinchoninic acid (BCA) in an alkaline environment and produced a blue-violet complex. As a result, the absorbance value that was measured by microplate reader was proportional to the protein concentration.

GOx/ChOx activity assay. The catalytic activity of the GOx/ChOx on the surface of the SMMB was evaluated by testing its ability to catalyse ABTS chromogenic reaction. Briefly, active GOx catalyzes the oxidation of glucose to gluconolactone, meanwhile O_2 is reduced to H_2O_2, which further oxidizes colorless ABTS to green $ABTS^{+}$ under the catalysis of HRP. After the reaction is over, the absorbance value was recorded by microplate reader.

S2 Characterization of SMMB with X-Ray Diffraction (XRD).

X-Ray Diffraction (XRD) was used to analyze the crystal phase structure of the SMMB. As shown in Fig S1, the typical diffraction peaks (2θ) appear at 31.76°, 34.42°, 36.25°, 47.53°, 56.59°, 62.85°, 66.37°, 67.94°, and 69.08° that correspond to the primary diffraction of the (100), (002), (101), (102), (110), (103), (200), (112), and (201) crystal planes, are consistent with the hexagonal wurtzite structure of ZnO, which indicates ZnO has been distributed on the surface of the MMB.

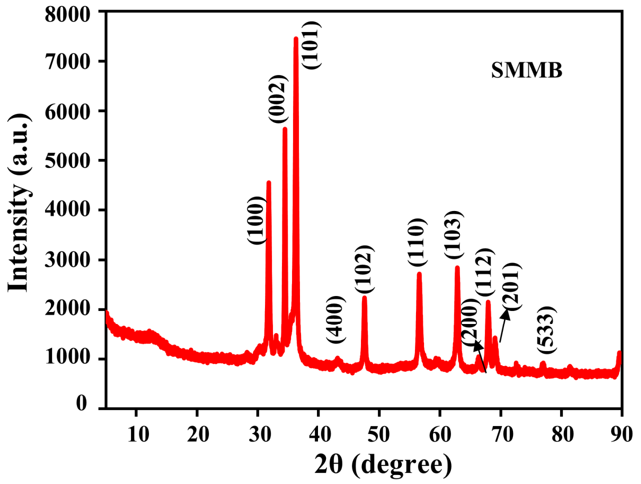

Figure S1. XRD pattern of SMMB.

S3 Characterization of SMMB with high-resolution transmission electron microscopy (HR-TEM).

Then, high-resolution transmission electron microscopy (HR-TEM) was also used to analyze the atomic-level structure of the MMB@ZnO and the SMMB. The images are shown in Figure S2 A&B, and the representative crystal regions that highlighted in red boxes display clear lattice fringes. The corresponding fast Fourier transforms (FFT, a1 and b1) and the inverse FFT (IFFT) as a2 and b2 were performed by mathematical Fourier transform. It clearly shows that the average interplanar spacings of MMB@ZnO and SMMB are 0.248 nm and 0.26 nm (a3 & b3), respectively, which are consistence with the (101) and (002) crystal planes of the wurtzite ZnO structure, and proves ZnO has grown on the surface of the MMB.

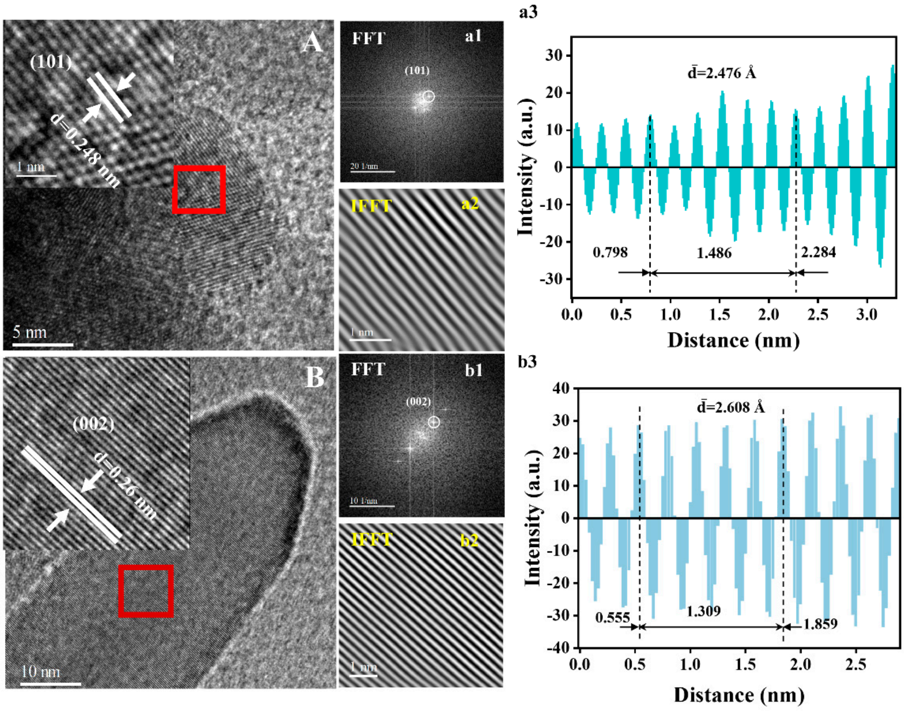

Figure S2. HR-TEM analysis of the MMB@ZnO and SMMB. (A) The HR-TEM image of MMB@ZnO. (a1) The FFT image of MMB@ZnO. (a2) IFFT image of MMB@ZnO. (a3) Intensity profiles measured from MMB@ZnO. (B) The HR-TEM image of SMMB. (b1) The FFT image of MMB@ZnO. (b2) IFFT image of MMB@ZnO. (b3) Intensity profiles measured from SMMB.

S4 Analysis of the GOx adsorption ability of different microbeads by microplate reader.

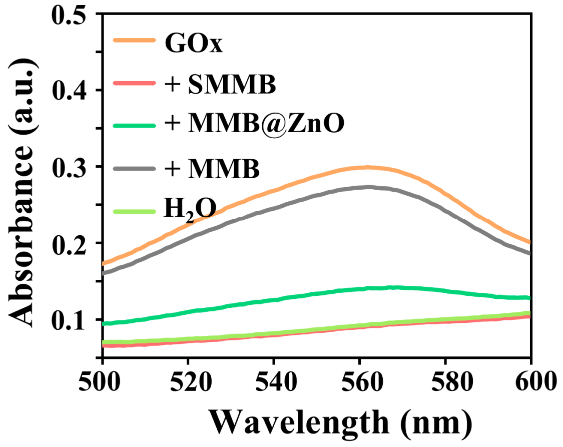

Figure S3. Comparison of the GOx adsorption ability of different microbeads. The concentration of the GOx and the microbeads were 500 μg/mL and 800 μg/mL, respectively.

S5 Analysis of the anchoring of GOx on SMMB by Fourier transform near infrared spectroscopy (FTIR).

To further confirm the anchoring of the GOx on SMMB, the SMMB and the GOx/SMMB were analyzed by FTIR, respectively. As presented in Figure S4, compared to the results of the solo SMMB, the measurement results of the SMMB/GOx showed an absorption peaks at 1590-1600 cm^{-1} corresponding to the bending vibrations of the N-H bonds, and an absorption peaks at 1590-1600 cm^{-1}, which is attributed to the stretching vibrations of the C=O bonds. It can be indicated that there are proteins on the SMMB surface.

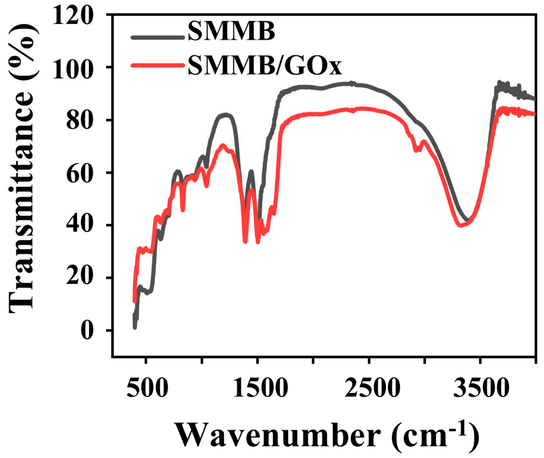

Figure S4. FTIR analysis results of the SMMB and the SMMB/GOx.

S6 Cyclic voltammetry (CV) profiles for various microbeads modified electrodes.

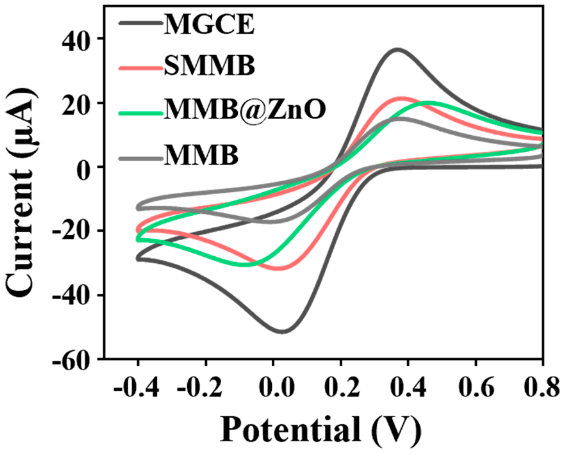

Figure S5. The CV curve of the different microbeads functionalized MGCE in 5 mM $[Fe(CN)_6]^{3-/4-}$ solution. Scan rate was 0.1 V/s.

S7 Comparison the GOx loading efficiency of MMB@ZnO and SMMB.

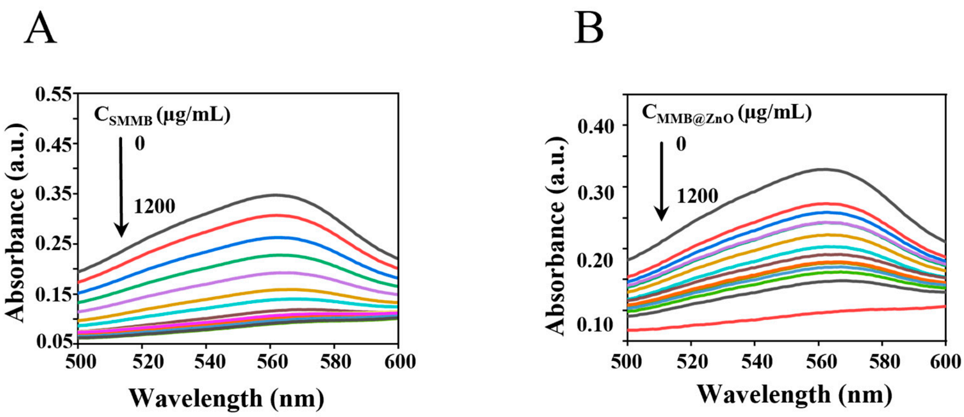

Figure S6. Comparison of the GOx adsorption ability by adding SMMB and MMB@ZnO with different concentrations into 500 μg/mL GOx solution respectively and quantifying the residual GOx by BCA Protein Assay Kit. (A) The absorbance detection results for the SMMB added sample. (B) The absorbance detection results for the MMB@ZnO added sample. The concentrations of the microbeads were 0, 100, 200, 300, 400, 500, 600, 700, 800, 900, 1000, 1100, 1200 μg/mL, respectively.

S8 Optimization of pH

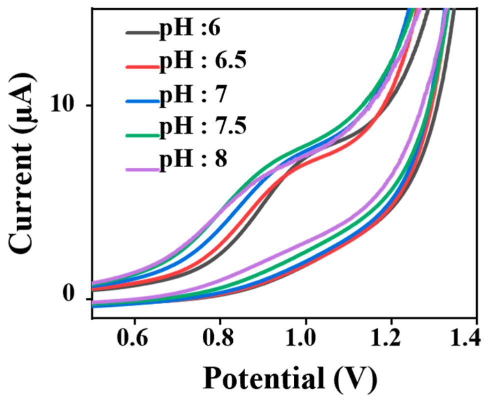

Figure S7. CV response of the SMMB-based biosensor in different pH solutions of phosphate-buffered saline (PBS) with 1.25 mM glucose.

S9 Performance of the SMMB-based electrochemical biosensor for glucose detection.

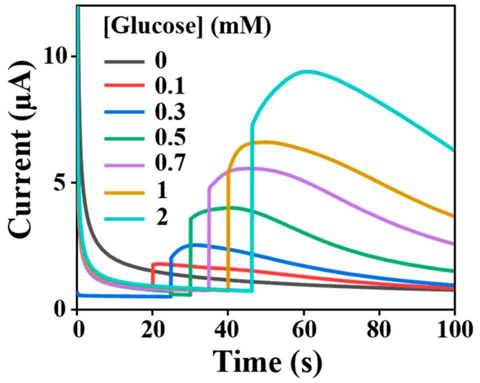

Figure S8. Analyzing the detection ability of the SMMB based electrochemical biosensor for different concentrations of glucose.

S10 Specificity analysis of the SMMB-based electrochemical biosensors

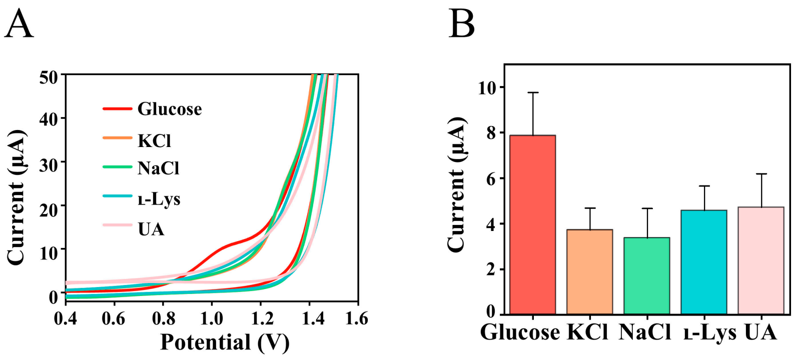

Figure S9. Specificity analysis of the SMMB-based electrochemical biosensors. (A) CV response of the SMMB-based electrochemical biosensor for different non-target biomarkers (KCl, NaCl, L-Lys and UA). (B) Analyze the oxidation current peak of the different samples (n=3). All of the biomarkers were 2.5 mM.

S11 Analysis of the sensitivity of the SMMB-based biosensor for detecting glucose in plasma.

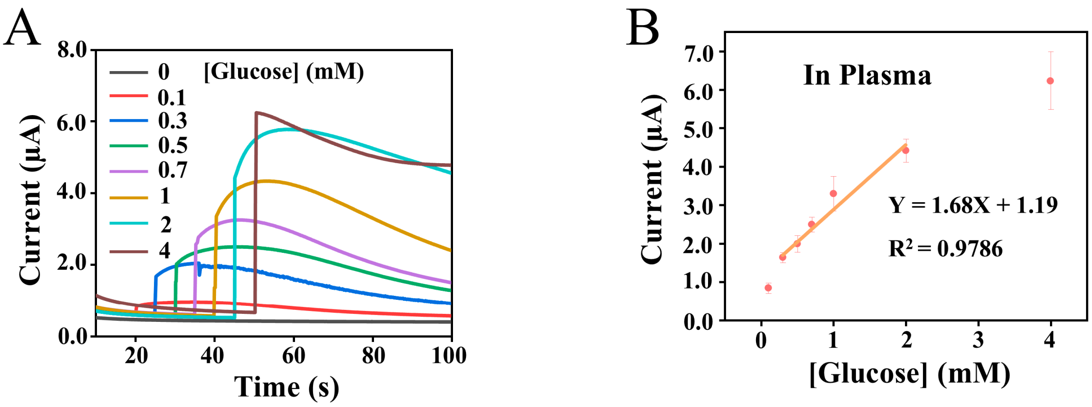

Figure S10. (A) Analyzing the detection ability of the SMMB-based electrochemical biosensor for different concentrations of glucose in 100× diluted plasma. (B) Analyzing the detection ability of the SMMB-based electrochemical biosensor for different concentrations of glucose in 100× diluted plasma.

S12 Analyze the anchoring activity of ChOx on SMMB.

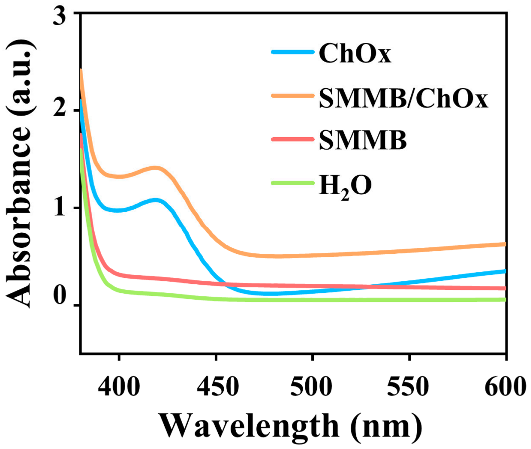

Figure S11. Verification of the activity of ChOx on SMMB by microplate reader.

S13 Analysis the ChOx loading ability of SMMB

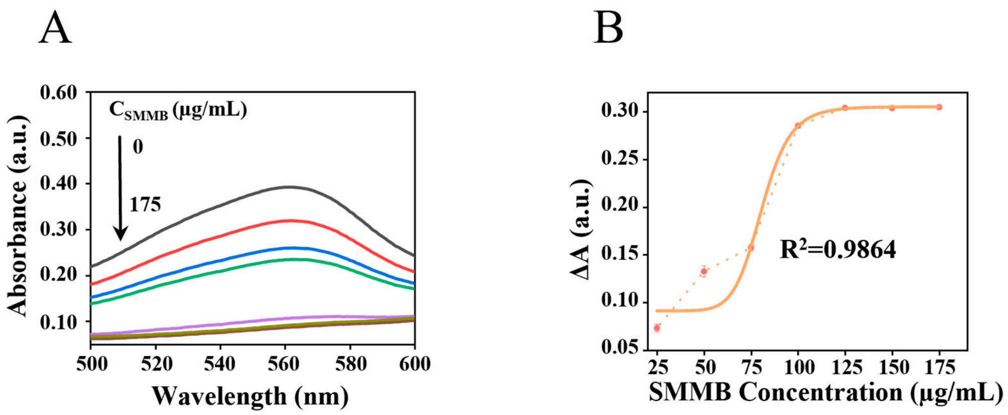

Figure S12. Analyzing the ChOx adsorption ability of the SMMB. (A) The absorbance detection result of the samples with different concentrations (0, 25, 50, 75, 100, 125, 150 & 175 μg/mL, respectively). The residual ChOx in supernatant was quantified by BCA kit. The concentration of the ChOx was 500 μg/mL. (B) The change in absorbance (ΔA) with the SMMB Concentration.

S14 The performance of the SMMB-based electrochemical biosensor for cholesterol (Chot) detection.

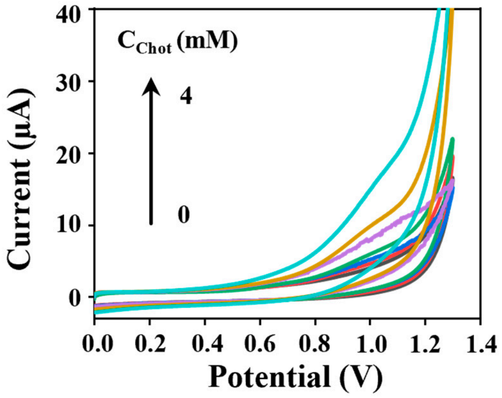

Figure S13. Analyzing the detection sensitivity by adding Chot with different concentrations (0, 0.3, 0.5, 0.7, 0.9, 2, 4 mM) into the system respectively.

Table S1. Existing electrochemical sensors for glucose detection§.

Electrode Materials	Sensitivity ($\mu A \cdot mM^{-1}\ cm^{-2}$)	LOD (μM)	Linear range (mM)	Ref.
CDs	--	250	1-12	[46]
PtNPs	42.43	120	0.12-10	[47]
ZnO–Au–Cu_2O	2.88	80	1-19	[48]
CGC	38.8	150	0.15-12	[49]
3D porous graphene aerogel@GOx	--	870	1-18	[50]
SMMB	73.32	100	0.1-1	This work

§ CDs: carbon dots. PtNPs: platinum nanoparticles. CGC: Crab gill–derived nanorod-like carbons.
